# Assessment of Text Documentation Accompanying Uncoded Diagnoses in Computerized Health Insurance Claims in Japan

**DOI:** 10.2188/jea.JE20140105

**Published:** 2015-03-05

**Authors:** Shinichi Tanihara

**Affiliations:** Department of Public Health and Preventive Medicine, School of Medicine, Fukuoka University, Fukuoka, Japan; 福岡大学医学部衛生公衆衛生学

**Keywords:** coding, health insurance claim, ICD-10, uncoded diagnoses

## Abstract

**Background:**

Uncoded diagnoses in health insurance claims (HICs) may introduce bias into Japanese health statistics dependent on computerized HICs. This study’s aim was to identify the causes and characteristics of uncoded diagnoses.

**Methods:**

Uncoded diagnoses from computerized HICs (outpatient, inpatient, and the diagnosis procedure-combination per-diem payment system [DPC/PDPS]) submitted to the National Health Insurance Organization of Kumamoto Prefecture in May 2010 were analyzed. The text documentation accompanying the uncoded diagnoses was used to classify diagnoses in accordance with the International Classification of Diseases-10 (ICD-10). The text documentation was also classified into four categories using the standard descriptions of diagnoses defined in the master files of the computerized HIC system: 1) standard descriptions of diagnoses, 2) standard descriptions with a modifier, 3) non-standard descriptions of diagnoses, and 4) unclassifiable text documentation. Using these classifications, the proportions of uncoded diagnoses by ICD-10 disease category were calculated.

**Results:**

Of the uncoded diagnoses analyzed (*n* = 363 753), non-standard descriptions of diagnoses for outpatient, inpatient, and DPC/PDPS HICs comprised 12.1%, 14.6%, and 1.0% of uncoded diagnoses, respectively. The proportion of uncoded diagnoses with standard descriptions with a modifier for *Diseases of the eye and adnexa* was significantly higher than the overall proportion of uncoded diagnoses among every HIC type.

**Conclusions:**

The pattern of uncoded diagnoses differed by HIC type and disease category. Evaluating the proportion of uncoded diagnoses in all medical facilities and developing effective coding methods for diagnoses with modifiers, prefixes, and suffixes should reduce number of uncoded diagnoses in computerized HICs and improve the quality of HIC databases.

## Abstract (ja)

**【背景】:**

診療報酬明細書（以後、レセプト）データにおける未コード化傷病名の存在はレセプトを用いた保健統計や研究においてバイアスをもたらす可能性が存在するが、未コード化傷病名が発生する原因は解明されていない。未コード化傷病名の特性を分析することで未コード化傷病名が発生する原因を検討することが本研究の目的である。

**【方法】:**

熊本県国保連合会に提出された2010年5月診療分の電子化されたレセプト（医科入院外、医科入院、診断群分類に基づく一日当たり包括評価方式（the diagnosis procedure-combination per-diem payment system、以後DPC/PDPS））に記載された全ての傷病名3,746,121件から未コード化傷病名を抽出し、それぞれに対応するテキスト傷病名をレセプト電算処理システムマスターファイルの「疾病及び関連保健問題の国際統計分類：International Statistical Classification of Diseases and Related Health Problems」の第10回修正版（ICD10）対応標準病名傷病名マスターと比較し、１）標準病名、２）標準病名＋修飾語、３）標準病名に一致しない傷病名、４）分類不能、の４種類に分類した。さらに、１～３）についてはICD10の大分類に分類した結果をレセプトの種類（医科入院、医科入院外、DPC/PDPS）別に集計し、レセプトの種類かつICD10大分類別に未コード化傷病名の種類を集計した。

**【結果】:**

全体で未コード化傷病名は363,753件（9.7%）認められた。標準病名に一致しない傷病名の割合は医科入院、医科入院外、DPC/PDPSでそれぞれ12.1%、14.6%と1.0%であった。未コード化傷病名に対応するテキスト傷病名をICD10大分類別に検討した結果、眼および付属器の疾患（ICD10:H00-H59）は標準病名＋修飾語に分類されたものの割合が最も高かった。

**【結論】:**

未コード化傷病名の出現パターンはレセプトの種類や傷病大分類によって異なっていた。全ての医療機関で未コード化傷病名の出現割合を評価することや、標準病名に修飾語が付与された場合のコード化についてより効果的な方法を開発することで未コード化傷病名の割合を減少させ、レセプトに記載された情報を用いたデータベースの質を向上させることが可能となる。

## INTRODUCTION

A precise evaluation of the burden of disease is required to set priorities in health policy. Health insurance claims (HICs) are prepared by healthcare providers for reimbursement of their services. In Japan, HIC records contain information about health insurance qualification status, healthcare costs, clinical procedures, and diagnoses. Statistics on medical expenditures in Japan, such as those reported in the Estimation of National Medical Expenditures, Social Insurance Claims Survey, and the National Health Insurance Medical Benefit Surveys, are based on HICs. The following list outlines healthcare indicators that recently have been calculated using HIC information: the quality of care of patients with diabetes,^1^ the completeness of the infectious disease surveillance system,^2^ the relationship between disease type and patients’ health-seeking behaviors,^3^ the relationships between health guidance for metabolic syndrome and outpatient charges and drug costs and metabolic syndrome,^4^ the association between hospital case volume and mortality in non-elderly pneumonia patients,^5^ and regional differences in the performance of bone marrow transplants.^6^ There are limitations in the information recorded in HICs in Japan due to regulations on medical cost reimbursement. First, most HICs contain more than one diagnosis^7^^–^^9^ because healthcare providers submit only one HIC describing all of the healthcare services provided to an individual during a calendar month. Previously, HICs were submitted on paper, and it was common to select only one principal diagnosis from a HIC for the database, even if there were multiple diagnoses. This practice resulted from the labor costs of inputting the information from the HIC into a database. It has been reported that hypertension tended to be selected as the principal diagnosis among the elderly insured by the National Health Insurance for Medical Services for the Aged.^7^ Thus, the estimation of disease-specific medical expenditures using HICs may be biased.^8^^,^^10^

In Japan, confirmed, unconfirmed, and disproved diagnoses are all included in HICs.^10^^,^^11^ Furthermore, health insurance coverage is based primarily on fee-for-service reimbursement, with regulations dictating that each clinical procedure be described and justified by a corresponding diagnosis. For certain diagnostic categories (eg, neoplasm), various examinations are usually conducted, including consultations, imaging, and investigations using tumor markers. When test results indicate that a suspected disease is not present, rule-out diagnoses are included in the HIC to ensure reimbursement for the clinical procedures conducted. Technical limitations prevent the assessment of all the information mentioned in a HIC; therefore, information about multiple and rule-out diagnoses have been ignored in estimates of disease-specific medical expenditures, which may introduce bias. For example, more than one-third of the medical expenditures for outpatient care of neoplasms are spent on rule-out diagnoses,^10^ and there are differences in procedures and medical expenditures for inpatients with and without a rule-out diagnosis of sepsis.^9^

Until recently, most of the technical limitations of using information contained in HICs were caused by HICs that were submitted on paper. The labor cost of maintaining a database containing all of the information on the claim forms prevented assessments of complete HICs. Therefore, the information about multiple and rule-out diagnoses in HICs were often ignored in estimates of disease-specific medical expenditures.^8^^–^^12^ After August 2010, however, all hospitals and medical clinics were mandated to submit computerized HICs to claim reimbursement for the cost of healthcare services. Thus, 96.6% of HICs were computerized by March 2014.^12^

Uncoded diagnoses in computerized HICs have been recognized as a new problem in the management of these data.^13^^,^^14^ Medical facilities are required to code all of the diagnoses according to the International Statistical Classification of Diseases and Related Health Problems, 10th Revision (ICD-10) before submitting the computerized HIC.^15^ However, if the medical facilities are unable to code a diagnosis, it is listed as “uncoded”; in this case, the HICs are submitted with text documentation related to the uncoded diagnoses. The proportion of uncoded diagnoses electronically submitted to National Health Insurance Organization (NHIO) of Kumamoto Prefecture in 2010 was reported as approximately 10%.^13^^,^^14^

Uncoded diagnoses are not used in the summaries of key health statistics, such as the Social Insurance Claims Survey and the National Health Insurance Medical Benefit Surveys. The proportion of uncoded diagnoses varies by the type of HIC and disease category, and uncoded diagnoses may have introduced bias into Japanese health statistics that are based on HICs.^14^ The proportion of uncoded diagnoses was relatively high for some diagnoses, especially *Diseases of the ear and mastoid process* and *Injury, poisoning, and certain other consequences of external causes*, which are supposed to be accompanied by information about the disease sites (eg, the right or left side of the body). The causes underlying uncoded diagnoses in computerized HICs must be identified to improve the accuracy of Japanese health statistics that are based on computerized HIC data, such as the estimation of disease-specific medical expenditures. The aim of this study is to investigate the causes of uncoded diagnoses in computerized HICs by analyzing the text documentation accompanying the uncoded diagnoses.

## METHODS

### Health insurance claims

This study reviewed all 363 753 uncoded diagnoses of the 3 804 246 diagnoses in HICs that were electronically submitted to the NHIO of Kumamoto Prefecture in May 2010. The details of HICs in Japan have been described in our previous report.^14^

Medical facilities are required to code all diagnoses according to the standard descriptions of diagnoses defined in the “Manual about Specifications of the Computerized Health Insurance Claim System Master File”^15^ before submitting HICs. The coding of the standard descriptions of diagnoses, which is defined in the master file, is based on the ICD-10. In addition, every diagnosis is accompanied by supporting text documentation. We first classified text documentation accompanying the uncoded diagnoses according to the disease categories in the ICD-10, which are subdivided into chapters. Next, we classified text documentation as follows: 1) a standard description of diagnoses, 2) a standard description with a modifier, 3) a non-standard description of diagnoses, and 4) unclassifiable text documentation. The “standard description of diagnoses” classification refers to text documentation that is identical to one of the standard descriptions. The “standard description with a modifier” classification is a combination of the standard description of diagnoses plus information, such as disease sites (eg, right or left side of the body). A “non-standard description of diagnoses” classification occurs when the description of the diagnosis in the text documentation is different from the “standard description” but can still be classified according to the ICD-10 categories. The “unclassifiable text documentation” classification refers to text documentation that cannot be classified in one of the other three categories. The latter two categories are classified according to the presence or absence of a modifier.

### Statistical analysis

The proportions of the types of text documentation accompanying the uncoded diagnoses from outpatient care, inpatient care, and DPC/PDPS were compared, and the diagnoses were classified according to the disease categories in the ICD-10. The proportions of uncoded diagnoses according to the type of text documentation and HIC were compared. Descriptive summary statistics are presented as frequencies and proportions for the categorical data. The χ^2^ test was used to compare the proportions of uncoded diagnoses among the disease categories and the overall proportion of uncoded diagnoses according to the type of HIC. A two-tailed *P* value of <0.05 was considered statistically significant. All analyses were performed using IBM SPSS Statistics, Version 19 (International Business Machines Corporation, Armonk, NY, USA).

### Ethical concerns

All personal information from the HIC data was deleted by the NHIO before the data were delivered to the researchers. This study was approved by the Institutional Review Committee of Fukuoka University.

## RESULTS

Table 1 shows the number of uncoded diagnoses analyzed according to the type of claim and type of accompanying text documentation. Among the 363 753 uncoded diagnoses included in the analyses, 316 151 (86.9%) were from outpatient medical HICs, 35 493 (9.8%) were from inpatient medical HICs, and 12 109 (3.3%) were from DPC/PDPS HICs. Standard descriptions of diagnoses were included with approximately one-third of the text documentations submitted with the uncoded diagnoses from outpatient (34.0%) and inpatient (35.7%) HICs; however, standard descriptions of diagnoses comprised more than half of the DPC/PDPS HICs (53.8%). The proportions of uncoded diagnoses with standard descriptions with a modifier were 46.3%, 39.2%, and 44.7% for the outpatient, inpatient, and DPC/PDPS HICs, respectively. The proportions of uncoded diagnoses with non-standard descriptions of diagnoses were 12.1% for outpatient HICs, 14.6% for inpatient HICs, and only 1.0% for DPC/PDPS HICs. The proportions of uncoded diagnoses with unclassifiable text documentation from outpatient and inpatient HICs were 7.6% and 10.4%, respectively; only 0.6% of uncoded diagnoses in DPC/PDPS HICs included unclassifiable text documentation. The proportion of the types of text documentation accompanying uncoded diagnoses differed significantly according to type of HIC.

**Table 1.** The number of diagnoses and uncoded diagnoses according to type of claim and type of text documentation accompanying uncoded diagnoses

	Type of HIC								

Type of diagnoses	Outpatient		Inpatient		DPC/PDPS		Total		*P*
Total diagnoses	3 393 106		325 968		85 172		3 804 246		
Uncoded diagnoses	316 151	9.3%	35 493	10.9%	12 109	14.2%	363 753	9.6%	<0.001
Types of text documentation accompanying uncoded diagnoses									
Standard descriptions of diagnoses	107 451	34.0%	12 663	35.7%	6515	53.8%	126 629	34.8%	<0.001
Standard descriptions with a modifier	146 529	46.3%	13 931	39.2%	5408	44.7%	165 868	45.6%	
Non-standard descriptions of diagnoses	38 217	12.1%	5196	14.6%	118	1.0%	43 531	12.0%	
Unclassifiable text documentation	23 954	7.6%	3703	10.4%	68	0.6%	27 725	7.6%	

DPC/PDPS, Diagnosis Procedure Combination/Per-Diem Payment System; HIC, health insurance claim.

Table 2 shows the proportion of uncoded diagnoses according to the type of text documentation accompanying the diagnoses in the outpatient HICs after the documentation data were classified into major disease categories. For every disease category, the proportions of uncoded diagnoses were significantly different from the overall proportion of the uncoded diagnoses. The proportion of uncoded diagnoses with the standard description for diagnoses was lowest in the category of *Pregnancy, childbirth, and the puerperium* (4.5%). However, only seven diagnoses in total fell into this category. The proportion and number of uncoded diagnoses related to *Diseases of the eye and adnexa* were 5.4% and 2082 respectively. The proportion of uncoded diagnoses with standard descriptions of diagnoses was the highest in the *Mental and behavioral disorders* category (72.2%), followed by the *Diseases of the respiratory system* category (69.3%).

**Table 2.** The proportion of uncoded diagnoses according to type of text documentation accompanying diagnoses in outpatient HICs

		Types of text documentation accompanying uncoded diagnoses						

ICD-10 major disease categories		Standard descriptions of diagnoses		Standard descriptions with a modifier		Non-standard descriptions of diagnoses		*P*
		Uncoded	(%)	Uncoded	(%)	Uncoded	(%)	
1	Certain infectious and parasitic diseases	2234	32.6%	3729	54.4%	895	13.1%	<0.001
2	Neoplasms	1498	15.2%	6774	68.7%	1583	16.1%	<0.001
3	Diseases of the blood and blood-forming organs and certain disorders involving the immune mechanism	732	42.1%	880	50.6%	128	7.4%	<0.001
4	Endocrine, nutritional, and metabolic diseases	9289	60.5%	5100	33.2%	969	6.3%	<0.001
5	Mental and behavioral disorders	10 030	72.2%	2995	21.6%	865	6.2%	<0.001
6	Diseases of the nervous system	7369	58.4%	4187	33.2%	1070	8.5%	<0.001
7	Diseases of the eye and adnexa	2082	5.4%	35 325	91.1%	1364	3.5%	<0.001
8	Diseases of the ear and mastoid process	1984	41.8%	2280	48.0%	487	10.3%	<0.001
9	Diseases of the circulatory system	17 848	49.5%	16 387	45.4%	1847	5.1%	<0.001
10	Diseases of the respiratory system	6104	69.3%	1712	19.4%	995	11.3%	<0.001
11	Diseases of the digestive system	14 346	42.6%	14 502	43.1%	4811	14.3%	<0.001
12	Diseases of the skin and subcutaneous tissue	4022	21.3%	10 002	53.0%	4830	25.6%	<0.001
13	Diseases of the musculoskeletal system and connective tissue	14 114	33.6%	21 467	51.1%	6427	15.3%	<0.001
14	Diseases of the genitourinary system	6227	47.3%	5169	39.3%	1760	13.4%	<0.001
15	Pregnancy, childbirth, and the puerperium	7	4.5%	24	15.4%	125	80.1%	<0.001
16	Certain conditions originating in the perinatal period	15	44.1%	8	23.5%	11	32.4%	<0.001
17	Congenital malformations, deformations, and chromosomal abnormalities	708	22.4%	1344	42.6%	1103	35.0%	<0.001
18	Symptoms, signs, and abnormal clinical and laboratory findings not elsewhere classified	6712	47.7%	4709	33.5%	2654	18.9%	<0.001
19	Injury, poisoning, and certain other consequences of external causes	2130	11.6%	9935	54.1%	6293	34.3%	<0.001
	Total	107 451	36.8%	146 529	50.2%	38 217	13.1%	

HIC, health insurance claim; ICD-10, the International Statistical Classification of Diseases and Related Health Problems, 10th Revision.

The number of uncoded diagnoses with unclassifiable text documentation was 23 954. Major disease category 20 (External causes of morbidity and mortality) was not found in outpatient HICs.

The proportion of uncoded diagnoses with the standard description with a modifier was the largest in the *Diseases of the eye and adnexa* category (91.1%); *Neoplasms* had the second largest proportion (68.7%), and *Pregnancy, childbirth, and the puerperium* (15.4%) had the lowest proportion; however, only 24 diagnoses were classified in the latter category. The proportion and number of uncoded diagnoses in the *Diseases of the respiratory system* category, which was the second least frequently represented disease in this text documentation category, were 19.4% and 1712, respectively.

The proportion of uncoded diagnoses with non-standard descriptions of diagnoses was highest for *Pregnancy, childbirth, and the puerperium* (80.1%), followed by *Congenital malformations, deformations, and chromosomal abnormalities* (35.0%). The lowest proportion of uncoded diagnoses with non-standard descriptions of diagnoses was in the *Diseases of the eye and adnexa* category (3.5%), followed by *Diseases of the circulatory system* (5.1%).

Table 3 shows the proportion of uncoded diagnoses according to the type of text documentation accompanying the diagnoses for inpatient HICs. Except for *Certain conditions originating in the perinatal period*, the proportions of uncoded diagnoses were significantly different from the overall proportion of uncoded diagnoses among all disease categories. The proportion of uncoded diagnoses with standard descriptions of diagnoses was highest for *Certain conditions originating in the perinatal period* (71.4%). However, there were only seven diagnoses in this disease category and the difference was not statistically significant. For *Mental and behavioral disorders*, the category with the second highest proportion of uncoded diagnoses with standard descriptions, the proportion was 68.9%; the number of diagnoses classified in this category was 2077. The proportion of uncoded diagnoses with the standard descriptions of diagnoses was the lowest for *Pregnancy, childbirth, and the puerperium* (9.5%). However, there were only 21 diagnoses in this disease category. For *Injury, poisoning, and certain other consequences of external causes*, which was the category with the second lowest proportion (10.8%), there were 311 diagnoses.

**Table 3.** The proportion of uncoded diagnoses according to type of text documentation accompanying diagnoses in inpatient HICs

		Types of text documentation accompanying uncoded diagnoses						

ICD-10 major disease categories		Standard descriptions of diagnoses		Standard descriptions with a modifier		Non-standard descriptions of diagnoses		*P*
		Uncoded	(%)	Uncoded	(%)	Uncoded	(%)	
1	Certain infectious and parasitic diseases	340	31.5%	562	52.1%	176	16.3%	<0.001
2	Neoplasms	173	18.0%	579	60.1%	211	21.9%	<0.001
3	Diseases of the blood and blood-forming organs and certain disorders involving the immune mechanism	173	40.0%	229	53.0%	30	6.9%	<0.001
4	Endocrine, nutritional, and metabolic diseases	1098	53.4%	757	36.8%	202	9.8%	<0.001
5	Mental and behavioral disorders	2077	68.9%	735	24.4%	204	6.8%	<0.001
6	Diseases of the nervous system	990	44.8%	1015	45.9%	205	9.3%	<0.001
7	Diseases of the eye and adnexa	225	28.4%	516	65.1%	52	6.6%	<0.001
8	Diseases of the ear and mastoid process	81	60.0%	38	28.1%	16	11.9%	<0.001
9	Diseases of the circulatory system	1749	43.5%	2010	50.0%	260	6.5%	<0.001
10	Diseases of the respiratory system	749	55.0%	412	30.2%	202	14.8%	<0.001
11	Diseases of the digestive system	1932	46.3%	1738	41.6%	504	12.1%	<0.001
12	Diseases of the skin and subcutaneous tissue	581	25.5%	1028	45.2%	666	29.3%	<0.001
13	Diseases of the musculoskeletal system and connective tissue	814	29.3%	1482	53.3%	484	17.4%	<0.001
14	Diseases of the genitourinary system	559	40.8%	534	39.0%	277	20.2%	<0.001
15	Pregnancy, childbirth, and the puerperium	2	9.5%	1	4.8%	18	85.7%	<0.001
16	Certain conditions originating in the perinatal period	5	71.4%	2	28.6%	0	0.0%	0.194
17	Congenital malformations, deformations, and chromosomal abnormalities	39	25.3%	70	45.5%	45	29.2%	<0.001
18	Symptoms, signs, and abnormal clinical and laboratory findings not elsewhere classified	765	37.2%	669	32.5%	623	30.3%	<0.001
19	Injury, poisoning, and certain other consequences of external causes	311	10.8%	1554	53.8%	1021	35.4%	<0.001
	Total	12 663	39.8%	13 931	43.8%	5196	16.3%	

HIC, health insurance claim; ICD-10, the International Statistical Classification of Diseases and Related Health Problems, 10th Revision.

The number of uncoded diagnoses with unclassifiable text documentation was 3703. Major disease category 20 (External causes of morbidity and mortality) was not found in inpatient HICs.

The proportion of uncoded diagnoses with the standard description with a modifier was highest for *Diseases of the eye and adnexa* (65.1%) and second highest proportion for *Neoplasms* (60.1%), which was the same as observed in the outpatient HICs. However, the proportions in both of these disease categories were lower than those in the outpatient HICs. The proportion of uncoded diagnoses with the standard description with a modifier was lowest for *Pregnancy, childbirth, and the puerperium* (4.8%). However, there were only 21 diagnoses in this category. The second lowest proportion was observed in the *Mental and behavioral disorders* category (24.4%), which consisted of 735 diagnoses.

The proportion of uncoded diagnoses with non-standard descriptions of diagnoses was highest for *Pregnancy, childbirth, and the puerperium* (85.7%), as was observed among outpatient HICs. Once again, however, the total number of diagnoses in this disease category was small (18). The category with the second highest proportion was *Injury, poisoning, and certain other consequences of external causes* (35.4%). No uncoded diagnoses with the non-standard descriptions of diagnoses were observed in *Certain conditions originating in the perinatal period*. There were seven diagnoses classified in this disease category and the difference was not statistically significant. The second lowest proportion was in the *Diseases of the circulatory system* category (6.5%), with 260 diagnoses.

Table 4 shows the proportion of uncoded diagnoses according to the type of text documentation accompanying the diagnoses in DPC/PDPS HICs. Except for *Diseases of the ear and mastoid process*, the proportions of uncoded diagnoses were significantly different from the overall proportion of uncoded diagnoses among all of the disease categories. The proportion of uncoded diagnoses with the standard descriptions of diagnoses was highest for *Pregnancy, childbirth, and the puerperium* (93.5%), followed by *Mental and behavioral disorders* (93.0%). There were fewer than 100 diagnoses in each of these two disease categories. However, in the disease category with the fourth highest proportion of uncoded diagnoses with standard descriptions of diagnoses, *Endocrine, nutritional, and metabolic diseases*, 545 (81.3%) diagnoses were accompanied by standard descriptions of diagnoses. The proportion of uncoded diagnoses with standard descriptions of diagnoses was the second lowest for *Diseases of the eye and adnexa* (27.2%) and lowest for *Injury, poisoning, and certain other consequences of external causes* (13.3%).

**Table 4.** The proportion of uncoded diagnoses according to type of text documentation accompanying diagnoses in DPC/PDPS HICs

		Types of text documentation accompanying uncoded diagnoses						

ICD-10 major disease categories		Standard descriptions of diagnoses		Standard descriptions with a modifier		Non-standard descriptions of diagnoses		*P*
		Uncoded	(%)	Uncoded	(%)	Uncoded	(%)	
1	Certain infectious and parasitic diseases	230	72.8%	83	26.3%	3	0.9%	<0.001
2	Neoplasms	1619	58.5%	1137	41.1%	11	0.4%	<0.001
3	Diseases of the blood and blood-forming organs and certain disorders involving the immune mechanism	201	77.6%	57	22.0%	1	0.4%	<0.001
4	Endocrine, nutritional, and metabolic diseases	545	81.3%	119	17.8%	6	0.9%	<0.001
5	Mental and behavioral disorders	93	93.0%	5	5.0%	2	2.0%	<0.001
6	Diseases of the nervous system	226	64.2%	123	34.9%	3	0.9%	<0.001
7	Diseases of the eye and adnexa	137	27.2%	367	72.8%	0	0.0%	<0.001
8	Diseases of the ear and mastoid process	21	42.0%	29	58.0%	0	0.0%	0.152
9	Diseases of the circulatory system	1067	60.4%	697	39.5%	2	0.1%	<0.001
10	Diseases of the respiratory system	471	65.5%	245	34.1%	3	0.4%	<0.001
11	Diseases of the digestive system	742	66.6%	352	31.6%	20	1.8%	<0.001
12	Diseases of the skin and subcutaneous tissue	84	39.6%	119	56.1%	9	4.2%	<0.001
13	Diseases of the musculoskeletal system and connective tissue	258	28.7%	631	70.2%	10	1.1%	<0.001
14	Diseases of the genitourinary system	251	70.1%	103	28.8%	4	1.1%	<0.001
15	Pregnancy, childbirth, and the puerperium	43	93.5%	2	4.3%	1	2.2%	<0.001
16	Certain conditions originating in the perinatal period	21	87.5%	3	12.5%	0	0.0%	0.005
17	Congenital malformations, deformations, and chromosomal abnormalities	27	77.1%	7	20.0%	1	2.9%	0.008
18	Symptoms, signs, and abnormal clinical and laboratory findings not elsewhere classified	281	76.8%	75	20.5%	10	2.7%	<0.001
19	Injury, poisoning, and certain other consequences of external causes	198	13.3%	1254	84.5%	32	2.2%	<0.001
	Total	6515	54.1%	5408	44.9%	118	1.0%	

DPC/PDPS, Diagnosis Procedure Combination/Per-Diem Payment System; HIC, health insurance claim; ICD-10, the International Statistical Classification of Diseases and Related Health Problems, 10th Revision.

The number of uncoded diagnoses with unclassifiable text documentation was 68. There were 58 125 diagnoses classified as Major disease category 20 (External causes of morbidity and mortality) in the DPC/PDPS HICs, but no diagnoses were uncoded diagnoses in this disease category.

The proportion of uncoded diagnoses with the standard description with a modifier was highest for *Injury, poisoning, and certain other consequences of external causes* (84.5%), followed by *Diseases of the eye and adnexa* (72.8%). The proportions of uncoded diagnoses with the standard description with a modifier for *Diseases of the eye and adnexa* were high in all three types of HICs. The proportion of uncoded diagnoses with the standard description with a modifier was lowest for *Pregnancy, childbirth, and the puerperium* (4.3%), followed by the *Mental and behavioral disorders* category (5.0%). However, few diagnoses were categorized in the three least frequently represented disease categories.

For almost every disease category, the proportions of uncoded diagnoses with non-standard descriptions of diagnoses were lower in outpatient than inpatient HICs. The highest proportion of uncoded diagnoses with non-standard descriptions of diagnoses was found for *Diseases of the skin and subcutaneous tissue* (4.2%). No diagnosis was found for *Diseases of the eye and adnexa*, *Diseases of the ear and mastoid process*, or *Certain conditions originating in the perinatal period*.

For all three types of HICs, the proportions of uncoded diagnoses with standard descriptions of diagnoses were highest for *Endocrine, nutritional, and metabolic diseases* and *Mental and behavioral disorders* and were lowest for *Diseases of the skin and subcutaneous tissue* and *Injury, poisoning, and certain other consequences of external causes*. The proportions of uncoded diagnoses with standard descriptions with a modifier were highest for *Neoplasms*, *Diseases of the eye and adnexa*, and *Injury, poisoning, and certain other consequences of external causes* and lowest for *Mental and behavioral disorders*; *Pregnancy, childbirth, and the puerperium*; and *Certain conditions originating in the perinatal period*. The proportions of uncoded diagnoses with non-standard descriptions of diagnoses were highest for *Diseases of the skin and subcutaneous tissue*; *Pregnancy, childbirth, and the puerperium*; *Congenital malformations, deformations, and chromosomal abnormalities*; and *Injury, poisoning, and certain other consequences of external causes.* The proportions of uncoded diagnoses with non-standard descriptions of diagnoses were lowest for *Diseases of the blood and blood-forming organs*, *Certain disorders involving the immune mechanism*, *Diseases of the eye and adnexa*, and *Diseases of the circulatory system*.

## DISCUSSION

The present study was the first in Japan to investigate the reasons for uncoded diagnoses in computerized HICs by analyzing the text documentation that accompanied the uncoded diagnoses. The three main findings were as follows: 1) the pattern of text documentation that accompanied uncoded diagnoses varied by type of HIC, 2) the proportions of uncoded diagnoses with standard descriptions of diagnoses with a modifier comprised approximately 40%–45% of all types of HICs, and 3) the proportions of uncoded diagnoses with standard descriptions of diagnoses with a modifier varied by disease category.

The proportions of uncoded diagnoses with non-standard descriptions of diagnoses and unclassifiable text documentation comprised approximately 10% of both outpatient and inpatient HICs and approximately 1% of DPC/PDPS HICs. Certification to provide DPC/PDPS care is granted only to hospitals that submit the required data using the format specified by the regulations on medical cost reimbursement. Thus, medical facilities certified for DPC/PDPS are motivated to use the standard descriptions of diagnoses. In comparison, there is no penalty for using non-standard descriptions of diagnoses for outpatient and inpatient HICs.

Approximately one-half of the uncoded diagnoses included standard description of diagnoses with a modifier. The proportions of uncoded diagnoses with standard descriptions of diagnoses with a modifier were relatively high in disease categories in which it is important to distinguish the affected sites on the body, such as *Diseases of the eye and adnexa*. Since the diagnostic codes for the Japanese computerized HICs do not distinguish such sites because they are based on the ICD-10, diagnoses with additional information, such as the right or left side of the body, might appear difficult to code. Development of methods with acceptable labor costs for medical facilities to code diagnoses accompanied by additional information is required to reduce the number of uncoded diagnoses. In addition, evaluating the proportion of uncoded diagnoses from each medical facility and using incentives and penalties might be an effective method of reducing numbers of uncoded diagnoses.

The proportions of uncoded diagnoses with standard descriptions of diagnoses with a modifier were relatively high in the categories of *Diseases of the eye and adnexa* and *Injury, poisoning, and certain other consequences of external causes*, and were relatively low for *Certain conditions originating in the perinatal period*; *Mental and behavioral disorders*; and *Pregnancy, childbirth, and the puerperium*. The proportion of uncoded diagnoses varied by the type of HIC and disease category.^14^ If there was a tendency for certain types of diagnoses requiring additional information regarding body sites to be uncoded, it may have created a bias in estimating disease-specific medical expenditures.^14^

Approximately one-third of the accompanying text documentation with uncoded diagnoses were classified as standard descriptions of diagnoses. As there is no penalty for submitting uncoded diagnoses, some medical facilities may lack the motivation to complete the coding. Hospitalization charges per day are determined according to the principal diagnosis, which may account for the high proportion of uncoded diagnoses with standard descriptions of diagnoses in the DPC/PDPS HICs. This reimbursement scheme may be an additional reason why medical facility staff may not be motivated to complete the coding. There are various types of diagnoses in DPC/PDPS claims, including 1) principal diagnosis, 2) most resource-intensive diagnoses, 3) diagnoses prompting hospitalization, 4) comorbidity present at the time of admission, and 5) complications developed in the course of hospitalization. Further research on the relationship between the types of diagnoses in DPC/PDPS claims and uncoded diagnoses is required.

There are some limitations in this study. First, I investigated information only from the computerized HICs. Therefore, the characteristics of the medical facilities and the validity of the diagnoses were not assessed directly. An evaluation of the characteristics of the medical facilities, the validity of the diagnoses, and the accuracy of coding of the diagnoses in the HICs requires additional information using medical charts^16^^–^^19^ or telephone interviews.^20^

Second, this study investigated only whether the standard descriptions of the diagnoses and modifier were included in the text documentation that accompanied the uncoded diagnoses. If a description currently used for a specific disease is not endorsed by a society of specialists, the description may not be used in the future and, therefore, will be classified as an abolished description. Such abolished description of the diagnoses will be deleted from the standard description of diagnoses after one year. From the results of this study, it is presumed that most of the modifiers are related to disease sites (eg, right or left side of the body). Modifiers other than disease sites, such as “severe,” are defined in the manual on specifications of the computerized HIC system master file.^15^ Further investigation of the effect of such changes in definitions on the standard descriptions of diagnoses and the type of modifier, prefix, and suffix is necessary.

Third, this study assessed HICs for reimbursement services provided only in May 2010. The current providers of health statistics in Japan, including The Social Insurance Claims Survey and National Health Insurance Medical Benefit Surveys, also used HIC data from May only. In the past, most of the technical limitations associated with data from HICs were because they were derived from paper submissions of HICs.^7^^,^^8^^,^^10^^,^^11^ This study reviewed HICs that were electronically submitted; it has been reported that 96.6% of HICs were computerized by March 2014.^12^ Investigation of electronic HICs over a longer period is necessary.

In conclusion, the pattern of uncoded diagnoses varied by the type of HIC and disease category. Most of the text documentation that accompanied uncoded diagnoses was classified using the standard descriptions of diagnoses defined by the Japanese reimbursement rules and the standard descriptions of diagnoses with a modifier. An evaluation of the proportion of uncoded diagnoses in all medical facilities and the implementation of effective methods for coding diagnoses using modifiers, prefixes, and suffixes should reduce the proportion of uncoded diagnoses in the computerized HICs in Japan and improve the quality of HIC databases.

## ONLINE ONLY MATERIAL

Abstract in Japanese.
