# Supplementary material for: Assessment of Text Documentation Accompanying Uncoded Diagnoses in Computerized Health Insurance Claims in Japan
Source: J Epidemiol. 2015 Mar 5;25(3):181–8. doi: 10.2188/jea.JE20140105 (PMC4340994; doi:10.2188/jea.JE20140105)
Supplement: Abstract in Japanese. [file je-25-181-s001.pdf]

電子レセプトにおける未コード化傷病名に対応づけされたテキスト傷病名の分析

谷原真一
福岡大学医学部衛生公衆衛生学

【背景】診療報酬明細書（以後、レセプト）データにおける未コード化傷病名の存在はレセプトを用いた保健統計や研究においてバイアスをもたらす可能性が存在するが、未コード化傷病名が発生する原因は解明されていない。未コード化傷病名の特性を分析することで未コード化傷病名が発生する原因を検討することが本研究の目的である。
【方法】熊本県国保連合会に提出された 2010 年 5 月診療分の電子化されたレセプト（医科入院外、医科入院、診断群分類に基づく一日当たり包括評価方式（the diagnosis procedure-combination per-diem payment system、以後 DPC/PDPS））に記載された全ての傷病名 3,746,121 件から未コード化傷病名を抽出し、それぞれに対応するテキスト傷病名をレセプト電算処理システムマスターファイルの 「疾病及び関連保健問題の国際統計分類：International Statistical Classification of Diseases and Related Health Problems」の第 10 回修正版（ICD10）対応標準病名傷病名マスターと比較し、１）標準病名、２）標準病名＋修飾語、３）標準病名に一致しない傷病名、４）分類不能、の４種類に分類した。さらに、１～３）については ICD10 の大分類に分類した結果をレセプトの種類（医科入院、医科入院外、DPC/PDPS）別に集計し、レセプトの種類かつ ICD10 大分類別に未コード化傷病名の種類を集計した。
【結果】全体で未コード化傷病名は 363,753 件（9.7%）認められた。標準病名に一致しない傷病名の割合は医科入院、医科入院外、 DPC/PDPS でそれぞれ 12.1%、14.6%と 1.0%であった。未コード化傷病名に対応するテキスト傷病名を ICD10 大分類別に検討した結果、眼および付属器の疾患 （ICD10:H00-H59）は標準病名＋修飾語に分類されたものの割合が最も高かった。
【結論】未コード化傷病名の出現パターンはレセプトの種類や傷病大分類によって異なっていた。全ての医療機関で未コード化傷病名の出現割合を評価することや、標準病名に修飾語が付与された場合のコード化についてより効果的な方法を開発することで未コード化傷病名の割合を減少させ、レセプトに記載された情報を用いたデータベースの質を向上させることが可能となる。

キーワード：コード化、診療報酬明細書（レセプト）、ICD10、未コード化傷病名
